# Spectrum of microarchitectural bone disease in inborn errors of metabolism: a cross-sectional, observational study

**DOI:** 10.1186/s13023-020-01521-6

**Published:** 2020-09-16

**Authors:** Karamjot Sidhu, Bilal Ali, Lauren A. Burt, Steven K. Boyd, Aneal Khan

**Affiliations:** 1 McCaig Institute for Bone and Joint Health, Cumming School of Medicine, University of Calgary, 3280 Hospital Drive NW, Calgary, Alberta T2N 4Z6 Canada; 2 Alberta Children’s Hospital Research Institute, Cumming School of Medicine, University of Calgary, 28 Oki Drive NW, Calgary, Alberta T3B 6A8 Canada; 3 Medical Genetics and Pediatrics, Cumming School of Medicine, Alberta Children’s Hospital, University of Calgary, 28 Oki Drive NW, Calgary, Alberta T3B 6A8 Canada

**Keywords:** Inherited metabolic disease, Rare disease, Lysosomal storage disorders, High-resolution peripheral quantitative compute tomography, Finite element analysis, Bone density

## Abstract

**Background:**

Patients diagnosed with inborn errors of metabolism (IBEM) often present with compromised bone health leading to low bone density, bone pain, fractures, and short stature. Dual-energy X-ray absorptiometry (DXA) is the current gold standard for clinical assessment of bone in the general population and has been adopted for monitoring bone density in IBEM patients. However, IBEM patients are at greater risk for scoliosis, short stature and often have orthopedic hardware at standard DXA scan sites, limiting its use in these patients. Furthermore, DXA is limited to measuring areal bone mineral density (BMD), and does not provide information on microarchitecture.

**Methods:**

In this study, microarchitecture was investigated in IBEM patients (*n* = 101) using a new three-dimensional imaging technology high-resolution peripheral quantitative computed tomography (HR-pQCT) which scans at the distal radius and distal tibia. Volumetric BMD and bone microarchitecture were computed and compared amongst the different IBEMs. For IBEM patients over 16 years-old (*n* = 67), HR-pQCT reference data was available and Z-scores were calculated.

**Results:**

Cortical bone density was significantly lower in IBEMs associated with decreased bone mass when compared to lysosomal storage disorders (LSD) with no primary skeletal pathology at both the radius and tibia. Cortical thickness was also significantly lower in these disorders when compared to LSD with no primary skeletal pathology at the radius. Cortical porosity was significantly greater in hypophosphatasia when compared to all other IBEM subtypes.

**Conclusion:**

We demonstrated compromised bone microarchitecture in IBEMs where there is primary involvement of the skeleton, as well as IBEMs where skeletal complications are a secondary outcome. In conclusion, our findings suggest HR-pQCT may serve as a valuable tool to monitor skeletal disease in the IBEM population, and provides insight to the greatly varying bone phenotype for this cohort that can be used for clinical monitoring and the assessment of response to therapeutic interventions.

## Background

Inborn errors of metabolism (IBEM) are a heterogeneous group of disorders caused by a defect in the synthesis, metabolism, transport, and/or storage of metabolites [1]. Today, more than 1000 IBEMs have been identified and include amino acid disorders, lysosomal disorders, transport and mineral disorders, and mitochondrial disorders [2]. Despite IBEMs being individually rare, the cumulative incidence of IBEMs worldwide is predicted to be 1 in 800 newborns, and IBEMs can present at any age; fetal stage to advanced age [2]. Clinical presentations are also highly diverse, both genetically and phenotypically [3]. Common symptoms include growth disturbances, endocrine dysfunction, neurological abnormalities, and musculoskeletal complications. Of all presenting symptoms, musculoskeletal complications inhibiting physical functioning have been mentioned to be the one of the most debilitating aspects of the disorder by parents of diagnosed children [4]. Imaging plays an important clinical role in diagnosis and evaluation of severity and activity of skeletal disease, as well as monitoring treatment effect [5].

Dual-energy X-ray absorptiometry (DXA) has been adopted for monitoring bone involvement in IBEM patients, primarily for its high clinical utility, short scan times, low radiation, and well-established reference data [6]. In the general population, DXA has been most commonly employed for the diagnosis and prognosis of osteoporosis, assessing fracture risk, and monitoring treatment effects [7, 8]. The femoral neck and lumbar spine are standard scan regions in mature adults, as fractures at these locations have been associated with high morbidity in adults [9]. However, the use of DXA in patients with IBEMs presents a number of challenges. First, DXA is a two-dimensional analysis of complex three-dimensional structures and therefore is highly sensitive to the size and geometry of the bone [10]. This is particularly problematic in IBEM patients who can often be of short stature, increasing the risk of underestimating BMD. Second, given that the femoral neck and lumbar spine are standard diagnostic sites for DXA and many IBEM patients are at greater risk for scoliosis and often have orthopaedic hardware implanted at these locations, there is a greater frequency of limitations in the ability to measure BMD at these sites. Third, DXA is unable to reliably detect fracture risk in pediatrics patients, as fragility fractures have been reported in children with measurements within the normal range [11]. Lastly, DXA is unable to provide information on bone microarchitecture and geometry, which have shown to play an important role in determining whole-bone strength [12, 13]. To advance the clinical management of skeletal complications in IBEMs, alternative imaging modalities that consider bone size and geometry and explore microarchitectural properties warrant further investigation.

High-resolution peripheral quantitative computed tomography (HR-pQCT) is a non-invasive, low radiation, *in vivo* bone imaging modality that scans at the distal limbs and provides an assessment of volumetric bone density and bone microarchitecture in the cortical and trabecular compartment. Given that HR-pQCT is based on three-dimensional imaging of bone, it is robust for use in short stature individuals because of the avoidance of confounding effects occurring in two-dimensional techniques such as DXA. HR-pQCT is well suited to cases of patients with orthopaedic hardware and scoliosis, as scans are being captured at the peripheral skeletal sites, rather than axial, where these issues often arise. To date, single case reports and case studies on individual IBEMs have been published examining bone microarchitecture using HR-pQCT. We and others have reported impaired bone density and microarchitecture when compared to healthy controls, with the exception of osteopetrosis, where bone density and microarchitecture is significantly elevated [14–21].

HR-pQCT allows the possibility for prediction of bone strength by measuring failure load using finite element techniques. The finite element method is the procedure of performing *in silico* biomechanical strength tests, such as compressive loading, on bone scan data generated by HR-pQCT and has been validated using mechanical testing on cadaveric samples [22, 23]. The primary output of these tests is failure load – the amount of load a bone can sustain before yielding. Failure load has been shown to provide higher acuity in fracture prediction than by examining BMD or microarchitectural measurements alone [24, 25], and serves as a convenient intrinsic summary of the complex microarchitectural phenotypes that are often encountered.

The objectives of this study were to 1) examine how density, microarchitecture, and failure load varied between different IBEMs, 2) compute Z-scores in adults using an age- and sex-matched healthy-reference cohort and, 3) compare participants with and without fracture on density, microarchitecture, and failure load measurements.

## Methods

### Study participants

Adult and pediatric patients being managed for their IBEM diagnosis at Inherited Metabolic Disorders Clinic (Alberta Children’s Hospital, Alberta, Canada) were offered participation in the study in addition to their standard clinical care. Participants were recruited between February 2007 to July 2019. All procedures were approved by University of Calgary’s Conjoint Health Research Ethics Board (REB#15-0271) and informed written paediatric or adult consent was obtained. At the time of the scan, mobility, fracture history, and other musculoskeletal comorbidities were noted, as well as any bone-altering drugs the patient was prescribed at the time of the scan. To overcome the challenge of a small sample size in each individual IBEM, IBEMs were categorized based on their underlying pathophysiology known from the historical context of the disease (Table 1).

**Table 1:** Categorization and characteristics of IBEM patients

IBEM Category	Abbreviation^**a**^	N	% Females	Age (SD)
Disorders of metabolism with restricted diets	Disorders with restricted diets	18	50%	27.3 (14.6)
Glycogen storage disease Ia (*n*=5)				
Galactosemia (*n*=4)				
Isovaleric acidemia (*n*=1)				
Phenylketonuria (*n*=1)				
Homocystinuria (*n*=2)				
Alkaptonuria (*n*=3)				
Methylmalonic acidemia (*n*=1)				
Cobalamin C deficiency (*n*=1)				
Lysosomal Storage Diseases (LSD) with no primary skeletal pathology	LSD (skeletal)	16	69%	42.1 (14.1)
Fabry disease (*n*=16)				
Osteopetrosis	Osteopetrosis	3	0%	24.1 (17.8)
Osteopetrosis (*n*=3)				
Disorders associated with decreased bone mass mineralization	Bone mineralization disorders	13	62%	26.3 (25.1)
Osteogenesis imperfecta (*n*=5)				
Juvenile osteoporosis (*n*=2)				
Osteoporosis (*n*=3)				
Amylogenesis imperfecta (*n*=2)				
Albright hereditary osteodystrophy (*n*=1)				
Hypophosphatasia	HPP	9	67%	26.3 (25.1)
Hereditary hypophosphatemic rickets with hypercalciuria (*n*=1)				
Hypophosphatasia (*n*=8)				
Lysosomal Storage Diseases (LSD) with primary secondary changes in bone architecture or skeletal growth	LSD (non-skeletal)	17	59%	31.3 (21.5)
Gaucher disease (*n*=13)				
Mucopolysaccharidosis type I (MPS I; *n*=1)				
Mucopolysaccharidosis type II (MPS II; *n*=1)				
Mucopolysaccharidosis type IV (MPS IV; *n*=1)				
Alpha-mannosidosis (*n*=1)				
Disorders of the nervous, muscular or skeletal system limiting mobility	Neuromuscular disorders	18	56%	39.5 (23.5)
Friedreich ataxia (*n*=1)				
Pelizaeus-Merzbacher disease (*n*=1)				
Multiple sclerosis (*n*=1)				
Cerebral palsy (*n*=1)				
Pompe disease (*n*=4)				
Spinal muscular atrophy type I (*n*=1)				
Mitochondrial disease (*n*=7)				
Dilated cardiomyopathy with ataxia (DCMA) syndrome (*n*=2)				

^a^The abbreviation is what the IBEM category will be referred to in text

### HR-pQCT measurements

Patients were scanned at the distal radius and distal tibia with the first-generation HR-pQCT (XtremeCT, Scanco Medical AG, Brüttisellen, Switzerland) using a standard human in vivo scanning protocol (60 kVp, 1,000 μA, 100 ms integration time). The participants’ limbs were immobilized using a carbon cast to minimize motion during the acquisition of the scan. To identify the region of interest, a two-dimensional X-ray scan was taken. Reference lines were manually placed at the mid-inclination tuberosity and at the plateau of the tibial endplate, for radius and tibia scans, respectively. Scans were then acquired at 9.5 mm (radius) and 22.5 mm (tibia) proximally to the reference line. Each HR-pQCT scan resulted in a 9.02 mm thick stack composed of 110 slices with a nominal isotropic resolution of 82 μm. The total scan time was less than 3 minutes per limb and resulted in a maximum effective dose of approximately 5 μSv per scan. All scans underwent two independent assessments for scan quality related to movement artifact and were graded on a scale of 1 (no motion) to 5 (severe blurring and discontinuities) [26]. Scans with a score of 4 or higher were excluded from analysis. Daily quality control assessments ranged from <1% root mean squared coefficient of variance for density to 4% root mean squared coefficient of variance for microarchitecture parameters in our laboratory [27]. Individuals analyzing the scans were blinded to the diagnosis of the patient.

Given that 82% of humans are right hand dominant [28], the left tibia and left radius were used for analysis. Where a previous fracture was reported on the left limb, the opposite limb was used for analysis. For density parameters, total BMD (TtBMD, mg HA/cm^3^), cortical BMD (CtBMD, mg HA/cm^3^), and trabecular BMD (TbBMD, mg HA/cm^3^) were measured and reported in units of mg/cm^3^ of hydroxyapatite (HA). Morphometric parameters of trabecular number (TbN, mm^-1^), trabecular thickness (TbTh, mm), trabecular separation (TbSp, mm), cortical thickness (CtTh, mm), and cortical porosity (CtPo, %) were measured.

A semi-automated contour was generated around the periosteal surface of the bone to separate it from the surrounding soft tissue. The cortical and trabecular bone were separated using a threshold-based algorithm. From standard morphologic analysis, TtBMD, TbBMD, TbTh, and TbSp were obtained [29]. TbN was determined using ridge-extraction methods [30]. To determine CtBMD, CtTh, and CtPo, an auto-segmentation algorithm was used [31, 32]. This auto-segmentation algorithm has been shown to be more robust and has higher reproducibility to the standard morphologic analysis for cortical analysis, especially when the cortical bone is thin [32].

### HR-pQCT and osteopetrosis

**Fig. 1:**
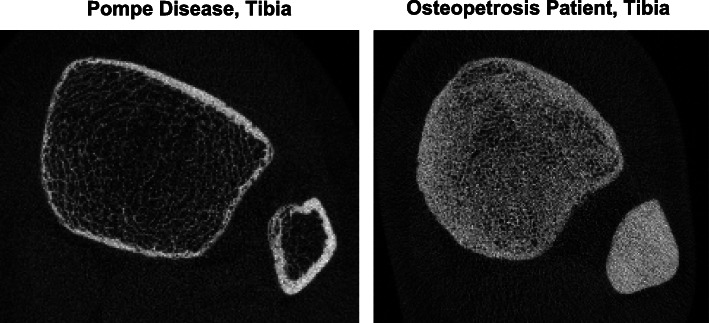
HR-pQCT imaging at the distal lower limb capturing the tibia and fibula of a Pompe patient (*left*) and a osteopetrosis patient (*right*)

When analyzing scans from patients diagnosed with osteopetrosis, the segmentation of cortical from trabecular compartments was difficult. An HR-pQCT image of an osteopetrosis patient and a patient with Pompe disease are shown in Fig. 1. It can be appreciated the discrimination of cortical bone from trabecular bone is markedly more difficult in the osteopetrosis patient when compared to the Pompe patient. For this reason, osteopetrosis patients were only examined for TtBMD, as cortical and trabecular-specific parameters may not be reliable.

### Finite Element (FE) analysis

Laplace-Hamming filtered and segmented HR-pQCT images were used to generate homogeneous finite element models. A linear uniaxial compression was then applied to radius and tibia models with 1% strain (Poisson’s ratio = 0.3, Young’s modulus = 6,829 MPa) [22]. The finite element models were solved using a custom large-scale finite element software (FAIM v8.0, Numerics88 Solutions, Alberta, Canada) and failure load in Newtons (N) was estimated based on the method proposed by Pistoia and colleagues, which was defined as the force required for a 2% critical volume to exceed 7000 critical μ-strain [33]. The precision for finite element is <4% root mean squared coefficient of variance in our laboratory [22].

### Statistical analysis

Statistical analyses were performed using IBM® SPSS® Statistics software (v26, IBM® SPSS® Inc., Chicago, Illinois, United States). Variables were checked for normality using Shapiro-Wilk tests and histograms. For variables that were not normally distributed, a log_10_ transformation was performed. Comparisons between IBEMs were explored using a one-way ANOVA with a Bonferroni adjustment. For participants 16 years of age and older, age- and sex-specific Z-scores were calculated from a healthy reference database [34].

Sub-analyses compared participants with and without a previous fracture using a Student’s t-test. Patients with osteopetrosis were excluded because these patients are predisposed to an increased risk of fractures due defective bone resorption that can lead to regional increases in BMD, whereas in other IBEMs, a high fracture risk is due to reduced BMD [35]. A Levene’s test for equality of variances was conducted.

For all statistical tests performed, two-sided *p* values below 0.05 were considered significant. Potential bone-altering confounders, such as medications, were not considered in our statistical calculations; however, a secondary analysis without inclusion of bone-altering drugs was performed and the findings were not different from our primary analysis, with some exceptions, which are reported in text.

## Results

### Participants

Table 1 displays baseline characteristics of the participants recruited. A total of 101 patients consented to participant, of which 58 were female. Participants ranged from the age of 4 to 81 years. Four participants were excluded due to severe motion artifacts on both radius and tibia HR-pQCT imaging sites. One participant was excluded due to HR-pQCT imaging erroneously capturing part of the growth plate for both the radius and tibia scan. Two participants were excluded because suspected diagnosis was not confirmed using genetic testing. Eighty-nine participants had acceptable radius and tibia scans (51 females, 35 males). Four participants had an acceptable tibia scan only (2 females, 2 males), and four participants had an acceptable radius scan only (4 males). Seven participants were actively on bone-alternating treatment at the time of the scan (5 on bisphosphonate, 1 on hormone replacement therapy, and 1 on both bisphosphonates and hormone replacement therapy). Fifteen participants reported previous axial and/or appendicular fracture, and of these participants 11 had reported more than one fracture.

### Comparison amongst IBEM categories

**Fig. 2:**
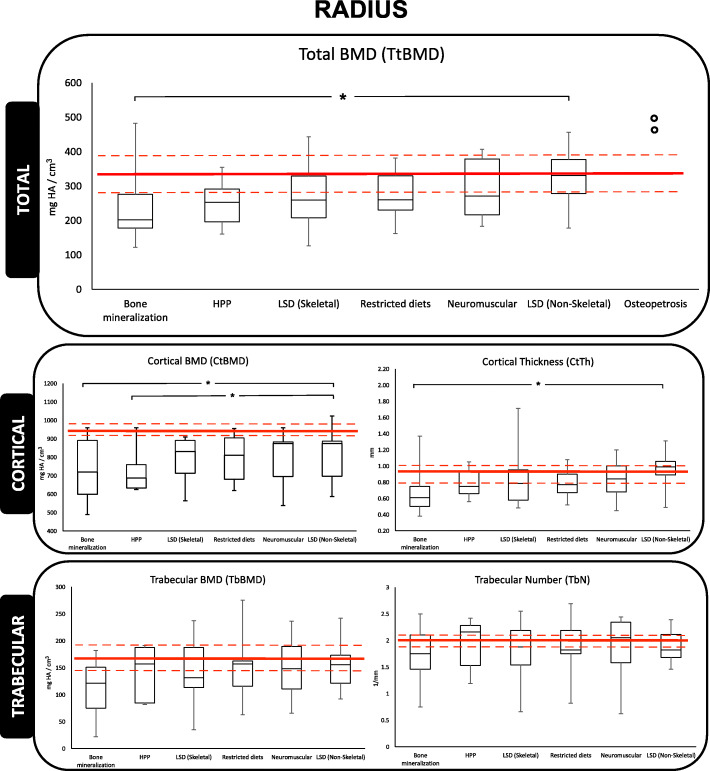
Radius analysis shown as box-and-whisker plot for HR-pQCT measured density and microarchitecture. Red lines, from top-to-bottom, represent 75^th^, 50^th^, and 25^th^ percentile in a reference population of young female adults aged 20-29 years-old [34]. Open points on total BMD (TtBMD) plot represent absolute values for osteopetrosis as there were not enough values to generate a box-and-whisker plot. Open square bracket with an asterisk (*) represents significant difference between two groups at an alpha value of 0.05

**Fig. 3:**
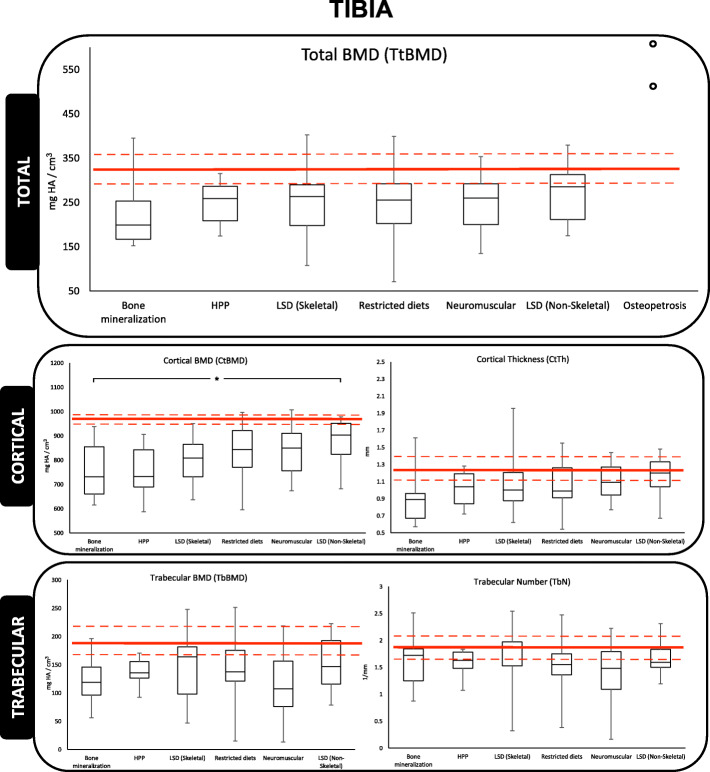
Tibia analysis for box-and-whisker plot for HR-pQCT measured density and microarchitecture. Red lines, from top-to-bottom, represent 75^th^, 50^th^, and 25^th^ percentile in a reference population of young female adults aged 20-29 years-old [34]. Open points on total BMD (TtBMD) plot represent absolute values for osteopetrosis as there were not enough values to generate a box-and-whisker plot. Open square bracket with an asterisk (*) represents significance difference between two groups at an alpha value of 0.05

**Fig. 4:**
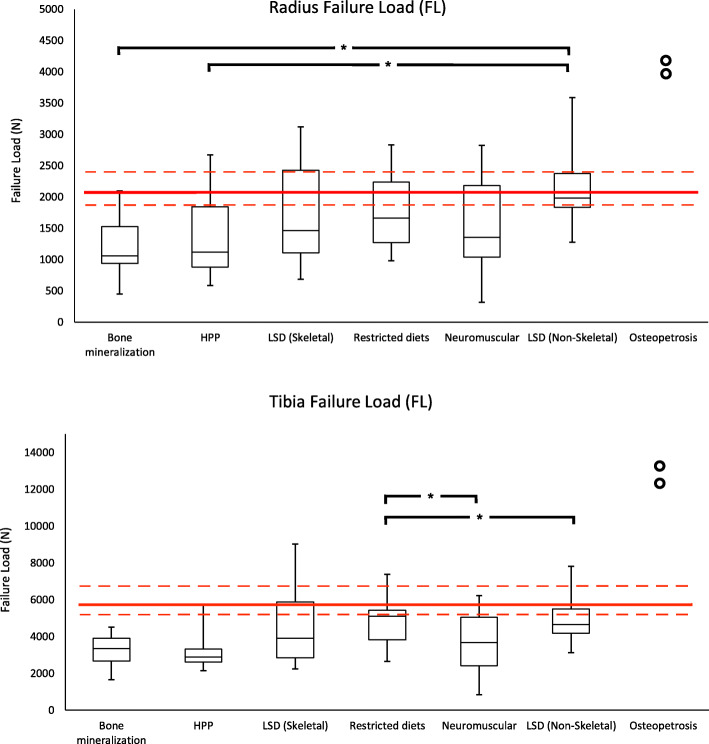
Box-and-whisker plot for HR-pQCT measured failure load in the radius (top) and tibia (bottom). Red lines, from top-to-bottom, represent 75^th^, 50^th^, and 25^th^ percentile in a reference population of young female adults aged 20-29 years-old [34]. Open points on total BMD (TtBMD) plot represent absolute values for osteopetrosis as there were not enough values to generate a box-and-whisker plot. Open square bracket with an asterisk (*) represents significance difference between two groups at an alpha value of 0.05

The median, interquartile range, minimum and maximum of the raw data is represented graphically in Figs. 2, 3 and 4 revealing that the outcome distributions were skewed Measurements not shown in these figures are provided in Additional file 1. The 25th, 50th, and 75th percentile lines of healthy young female adults (aged 20-29 years-old) are included on the figure for reference [34].

At both the radius and tibia skeletal sites the bone microarchitecture was generally poor in this population. In the radius, the median TtBMD, CtBMD and TbBMD of all IBEMs, with the exception of osteopetrosis, fell below the 50th percentile reported for healthy young female adults. Additionally, CtBMD of all IBEMs fell below the 25^th^ percentile when compared to healthy young female adults. Contrary to CtPo and TbSp measurements in the radius, which fell above the 25^th^ percentile. The TtBMD and CtTh of bone mineralization disorders was significantly lower compared to LSD (non-skeletal; *p*=0.025 and *p*=0.009, respectively). The CtBMD of both bone mineralization disorders and HPP was significantly lower than LSD (non-skeletal) participants (*p*=0.009 and *p*=0.013, respectively). The CtPo in HPP participants was significantly higher in comparison to restricted diets and LSD (non-skeletal) participants (*p*=0.034 and *p*=0.002, respectively). However, secondary analysis with the removal of participants with bone-altering drugs revealed CtPo of HPP to be significantly higher from LSD (non-skeletal; *p*=0.003) participants, only. Contrary to the cortical bone compartment, no significant findings were reported in the trabecular compartment measurements in the radius. In the tibia, TtBMD, CtBMD, and TbBMD all fell below the 25^th^ percentile compared to healthy young female adults. The CtBMD of participants with bone mineralization disorders was significantly lower when compared to LSD (non-skeletal; *p*=0.039). Similarly, as in the radius, no significant differences were found in the trabecular component. As expected, the absolute values of the two osteopetrosis measurements for TtBMD were largest in comparison to all other IBEM participants’ absolute values at both the radius and tibia.

Figure 3 presents the failure load of the radius and tibia across the IBEMs, and were consistent with the microarchitectural features already noted. In the radius and tibia, failure load in all IBEMs’, with the exception of osteopetrosis participants, fell below the 50th percentile for healthy young female adults, and in the tibia all IBEMs fell below the 25th percentile. In the radius, a significant difference was found between bone mineralization disorders and LSD (non-skeletal; *p*=0.003), as well as HPP and LSD (non-skeletal; *p*=0.040). In the tibia, neuromuscular disorders were significantly lower than LSD (non-skeletal) and disorders with restricted diets (*p*=0.047 and *p*=0.035, respectively).

### Z-Score calculation from normative data

For 67 of the 94 participants that were above the age of 16 years-old, a Z-score was calculated from available reference data (Table 2). At both the radius and tibia, all IBEMs had a negative Z-score for all density and microarchitecture measurements, with the exception of CtPo and TbSp, where the majority of the IBEMs had positive Z-scores. Osteopetrosis was an exception and had a positive TtBMD Z-score at the radius, +2.39. The Z-score for HPP was significantly larger in comparison to all other IBEMs (*p*<0.001) at the radius, and it was significantly larger than restricted diets and LSD (non-skeletal) at the tibia (*p*=0.043 and *p*=0.012, respectively). However, a secondary analysis with the removal of participants prescribed bone-altering drugs only revealed a significantly difference between HPP and LSD (non-skeletal) at the tibia (*p*=0.014). For estimated bone strength, bone mineralization disorders and disorders with restricted diet had significantly lower failure load at the radius in comparison to LSD (non-skeletal; *p*=0.003 and *p*=0.032, respectively).

**Table 2:** Calculation of Z-score^f^ for IBEM participants over the 16 years of age with age- and sex-matched controls

IBEM Category	TtBMD	CtBMD	TbBMD	CtTh	CtPo	TbN	TbTh	TbSp	FL
*Radius*									
Bone mineralization	-1.31	-2.28	-2.31	-0.54	0.26	-2.22	-1.80	4.22	-2.50^b^
HPP	-1.28	-3.40	-1.11	-0.88	8.24^**a**^	-1.21	-1.08	1.42	-0.86
LSD (skeletal)	-0.87	-2.05	-0.86	-0.87	1.00	-0.65	-0.64	1.31	-0.62
Restricted diets	-0.97	-2.07	-1.01	-1.08	0.92	-0.97	-0.52	1.59	-1.66^c^
Neuromuscular	-0.36	-1.98	-0.61	-0.15	1.86	-0.48	-0.47	1.71	-1.17
LSD (non-skeletal)	-0.18	-0.90	-0.43	0.12	0.00	-0.62	0.66	0.70	-0.02^b,c^
Osteopetrosis	+2.39	--	--	--	--	--	--	--	2.34
*Tibia*									
Bone mineralization	-1.39	-2.11	-2.13	-0.88	1.55	-1.65	-1.13	3.11	-2.49
HPP	-1.12	-2.52	-0.82	-1.20	3.75^d,e^	-0.95	0.00	0.94	-1.24
vLSD (skeletal)	-0.94	-1.85	-0.77	-0.75	1.58	-0.51	-0.21	2.36	-1.11
Restricted diets	-1.16	-1.47	-1.71	-0.94	0.36^d^	-1.07	-1.12	1.28	-1.93
Neuromuscular	-0.94	-1.22	-1.82	-0.61	0.41	-1.58	-0.80	6.78	-2.12
LSD (non-skeletal)	-0.49	-0.33	-0.70	-0.41	-0.24^e^	-0.85	-0.18	0.96	-0.83
Osteopetrosis	--	--	--	--	--	--	--	--	--

^a^HPP are significantly different to all other IBEM categories (with the exception of osteopetrosis), *p*<0.001

^b^Bone mineralization are significantly different to LSD (non-skeletal), *p*=0.003

^c^Restricted diets are significantly different to LSD (non-skeletal), *p*=0.032

^d^HPP are significantly different to restricted diets, *p*=0.043

^e^HPP are significantly different to LSD (non-skeletal), *p*=0.012

^f^Z-scores were calculated from published reference data [34]

### Fracture versus no fracture comparison

Fifteen participants reported a fracture in either the axial or appendicular skeleton prior to the acquisition of the scan. One of these participants was diagnosed with osteopetrosis and was excluded from statistical analysis. One of the participants had a radius scan, only, and another participant had a tibia scan, only. Half of these participants were under the age of 18 years-old. Table 3 presents the comparison between IBEM participants with and without a previous fracture. For both the radius and tibia, all density, microarchitecture and strength measurements were poorer in participants who suffered a fracture in comparison to participants who did not. In the radius, a significant difference was found in TtBMD, CtBMD, CtTh, and CtPo between individuals with fracture and those with no history of fracture (*p*<0.000, *p*=0.010, *p*=0.033, and *p*=0.006 respectively). A secondary analysis without inclusion of bone-altering drugs also captured significance in an additional measurement, TbBMD, between fracture (149.9±45.1 mg HA/cm^3^) and non-fracture (116.2±53.0 mg HA/cm^3^) participants at the radius (*p*=0.022). At the radius, TbTh was also significantly different between the fracture and no fracture group (*p*=0.009), however, the secondary analysis revealed no significant differences between these two groups (*p*=0.407). For the tibia, TtBMD was again significantly higher in the group with no fractures (*p*=0.012). Both density and microarchitecture in the cortical bone of the tibia, CtBMD, CtTh and CtPo, were all significantly different between the fracture and non-fracture group (*p*=0.011, *p*=0.002, and *p*=0.035, respectively). Though not statistically significant, failure load was 27.5% and 19.0% lower in the fracture group at the radius and tibia, respectively, compared to the non-fracture group. There was no significant difference between the mean age of the two groups.

**Table 3:** Comparison of IBEM participants without a previous fracture to IBEM participants with a previous fracture^a^

	No Fracture *N* = 75	Fracture *N* = 13	*p* value (α = 0.05)
*Radius*			
Age (years)	33.3 (19.6)	30.6 (25.3)	0.712
TtBMD (mg HA/cm^3^)	290.4 (81.8)	211.4 (53.5)	**<0.000**
CtBMD (mg HA/cm^3^)	816.1 (126.5)	714.2 (109.6)	**0.010**
TbBMD (mg HA/cm^3^)	147.1 (47.1)	121.3 (53.9)	0.077
CtTh (mm)	0.84 (0.25)	0.68 (0.16)	**0.033**
CtPo (%)	2.90 (1.94)	6.22 (5.76)	**0.006**
TbN (mm^-1^)	1.86 (0.45)	1.73 (0.51)	0.855
TbTh	0.07 (0.00)	0.06 (0.00)	**0.009**^a^
TbSp (mm)	0.53 (0.24)	0.59 (0.26)	0.263
Failure Load (N)	1714.5 (707.2)	1299.9 (640.7)	0.051
*Tibia*			
Age (years)	34.0 (19.4)	26.0 (24.1)	0.192
TtBMD (mg HA/cm^3^)	258.5 (71.4)	205.5 (45.6)	**0.012**
CtBMD (mg HA/cm^3^)	832.0 (103.5)	751.8 (93.9)	**0.011**
TbBMD (mg HA/cm^3^)	138.9 (51.4)	120.9 (41.9)	0.235
CtTh (mm)	1.10 (0.26)	0.86 (0.17)	**0.002**
CtPo (%)	5.50 (3.79)	8.27 (6.01)	**0.035**
TbN (mm^-1^)	1.58 (0.48)	1.53 (0.39)	0.918
TbTh	0.07 (0.00)	0.06 (0.00)	0.242
TbSp (mm)	0.75 (0.86)	0.63 (0.21)	0.932
Failure Load (N)	4278.3 (1564.3)	3536.4 (1399.5)	0.113

^a^Data are presented as mean and standard deviation in brackets. Bold font identifies significant difference

^a^A secondary analysis, removing participants on bone-altering drugs, revealed no significant difference between the no fracture and fracture group for TbTh (*p*=0.407)

## Discussion

In this study, we surveyed bone density and microarchitecture across different rare IBEMs using HR-pQCT. In addition to defining microarchitectural characteristics, bone strength was estimated by employing finite element methods and quantifying failure load from compressive loading simulations. IBEMs where HR-pQCT has been previously implemented to investigate bone quality include Gaucher disease, Pompe disease, Fabry disease, HPP, osteopetrosis, and osteogenesis imperfecta [14–21]. However, this is the first study to examine bone density and microarchitecture in a wide range of IBEMs, as well as employ finite element methods to predict bone strength. In our investigation, despite a distribution of density and microarchitecture values across the IBEMS, all disorders had impaired cortical and trabecular bone in comparison to a reference population. Significant differences were found between bone mineralization disorders and LSD (non-skeletal), as well as HPP and LSD (non-skeletal). Lastly, bone was compromised to large extent in IBEM participants who had sustained a fracture when compared to IBEM participants with no fracture history.

In both the radius and tibia, cortical and trabecular bone were largely compromised in comparison to young, healthy female adults, with the exception of osteopetrosis. Osteopetrosis density measurement were elevated in comparison to reference data. These findings are consistent with what has been previously reported in examining bone microarchitecture in sub-types of IBEMs using HR-pQCT [14–21]. Both cortical and trabecular bone play a crucial role in maintaining bone integrity and deficits in each have been shown to increase fracture risk [24, 36, 37]. Cortical density in bone mineralization disorders and HPP was significantly lower compared to LSD (non-skeletal), and total density was also significantly lower in bone mineralization disorders compared to LSD (non-skeletal). Cortical thickness was also significantly compromised in bone mineralization disorders compared to LSD (non-skeletal). Cortical porosity was significantly higher in HPP when compared to all other IBEM subtypes. Though more work is required to establish the determinants of bone loss in these IBEM subtypes, these observed differences may in part be explained by the disease pathophysiology. The IBEMs categorized into bone mineralization disorders and HPP, both have an underlying pathophysiology in which bone is directly affected by disease processes, whereas in LSD (non-skeletal), bone is often a secondary health outcome. For example, in osteogenesis imperfecta and juvenile osteoporosis, categorized under bone mineralization disorders, bone resorption is up-regulated and bone formation is down-regulated, disrupting bone remodelling processes and resulting in low bone mass [38, 39]. Whereas in Fabry disease, a LSD (non-skeletal) disorder, there is an accumulation of globotriaosylceramide that primarily affects small blood vessels, the heart, and the kidneys. Literature on bone involvement in Fabry disease is very limited and the bone involvement that has been reported is believed to be due to solid organ transplant, chronic renal failure, and steroid therapy, all factors that have been shown to influence bone health independently [40–43]. Therefore, we expected diseases with direct skeletal implications to have more compromised bone compared to those where the skeletal effects on bone is secondary.

Impairments in bone density and microarchitecture are reflected in measured failure load. Failure load at both the radius and tibia were lower in comparison to a healthy young cohort, but the radius is particularly affected. This may be explained by the protective effect of weight bearing in the tibia as weight bearing plays a major role in maintaining bone strength [44]. In the radius, the failure load of bone mineralization disorders and HPP was significantly lower when compared to LSD (non-skeletal). This was expected due to the severe deterioration of density and microarchitecture measured in the former IBEMs compared to LSD (non-skeletal). However, an unexpected finding was that the failure load in the tibia of neuromuscular disorders was significantly lower compared to disorders with restricted diets, as well as LSD (non-skeletal). Moreover, in adults, disorders with restricted diets had significantly lower failure load compared to LSD (non-skeletal). Ambulation was not considered in our statistical analysis and 6 of the 9 participants who had mobility impairments were diagnosed with a neuromuscular disorder. This may explain why bone strength was compromised in neuromuscular disorders to larger extent. For IBEMs with restricted diets, impairments to bone density and microarchitecture may be a result of nutritional deficiencies that can accompany strict diet restrictions that require lifelong adherence. Thus, treatment strategies in these disorders should consider the implications of these diets on bone health.

A subset of the cohort (*n* = 67), participants aged 16 years or over, were compared to a healthy sex- and age-matched cohort and their Z-scores were calculated. With the exception of osteopetrosis, all IBEMs had negative Z-scores at both the radius and tibia for bone density, bone microarchitecture, and failure load. These findings signify the prevalence of bone disease amongst all IBEM patients, regardless of whether skeletal complications are the primary or secondary outcome of the underlying disease pathophysiology.

Participants with previous fractures had significantly lower total bone density, cortical density and cortical thickness compared to those with no previous fracture in the radius. Cortical porosity was significantly greater in those with the previous fracture in comparison to those with no previous fractures. Similar results were found in the tibia. These findings are generally consistent with the literature, but interestingly, the affected compartments are different to extensive work done by others comparing post-menopausal women with and without fracture. Generally, these women who fracture have significantly lower overall density and cortical thickness, but cortical density was not significantly different between the two groups [24, 36]. Trabecular density and microarchitecture has been also reported to be significantly different between the fracture and non-fracture groups in these investigations. Similar findings to post-menopausal women have been reported in elderly men [37]. However, in our cohort, we primarily found significant differences in cortical bone between fracture and non-fracture groups. This suggests patterns of bone loss in IBEM participants may be different from adults with age-related bone loss, and specifically that cortical bone plays a key role in predisposing IBEM patients to fractures and should be carefully monitored, especially when a new treatment regime is initiated. The ability to measure the cortical compartment independently from the rest of the bone is well suited to HR-pQCT technology. Treatments shown to have positive effects on cortical bone include combination therapy with teriparatide and denosumab, as well as denosumab alone, whereas bisphosphonates have shown to primarily alter trabecular bone microarchitecture [45–48].

Estimated failure load was not significantly different between IBEM participants with and without a previous fracture and this is in contrast to the findings of others who showed it to be a strong and independent predictor of fracture [24, 36, 37, 49]. The trend was toward lower bone strength in our participants who suffered a previous fracture, which is consistent with the findings of others, and it may simply be that we did not have the power to detect a significant difference given the relatively small number of participants with a fracture in our cohort. However, it should be noted that most other studies are focused on the assessment of osteoporosis, where the main age-related changes to bone are dominated by compromised architecture. In our cohort, the tissue properties of bone itself may also play an important role, and a limitation of HR-pQCT is the ability to detect alterations in tissue mineralization. For example, in HPP, where bone is under-mineralized compared to healthy bone [50]. Therefore, caution should be used for estimated failure load when tissue properties are abnormal in the bone phenotype.

Some limitations of this study include our identification of the skeletal site for scanning by using a fixed distance scanning protocol, whereas using a relative site has been suggested as an alternative for pediatric scanning [51]. Similarly, while normative HR-pQCT data exists for children [51], we were unable to compare our paediatric participants to the published reference data due to differences in scan placement methodology. It is worth noting that when this study was first initiated in the 2007, there was no agreed upon paediatric approach for HR-pQCT scanning of children. Another challenge as mentioned previously is that estimated failure load assumes normal tissue constitutive properties, and this assumption is likely violated in some IBEM disorders. Our participants with previous fractures included all types of fractures pooled together (skeletal sites were not distinguished), and was a relatively small sample size. Finally, we did not measure serum vitamin D levels, and deficiency could alter bone density independently of IBEM diagnosis.

## Conclusions

Bone complications, particularly fractures, can often be the most debilitating aspect of a disorder due to the high morbidity and mortality associated with them [52]. A high risk of fractures has been reported in IBEMs where there is primary involvement of the skeleton, such as HPP and osteogenesis imperfecta. There are important limitations of using DXA as a basis for measuring bone density, and HR-pQCT is an emerging alternate technology that provides three-dimensional assessment of bone microarchitecture and associated bone strength that is particularly useful in these complex bone phenotypes. In the IBEM population we demonstrated that there is suboptimal bone quality at both the radius and tibia, with a greater burden on cortical bone, which can help inform future treatment approaches. Models of finite element analysis show these microarchitectural changes may result in fracture as lower failure load was estimated in IBEMs. In conclusion, we suggest that HR-pQCT provides unique insight into a personalized assessment of bone microarchitecture and associated failure load.

## Supplementary information

**Additional file 1: Supplemental Figure 1.** Additional HR-pQCT measurements plotted. Radius and tibia analysis shown as box-and-whisker plot for HR-pQCT measured microarchitecture. Red lines, from top-to-bottom, represent 75^th^, 50^th^, and 25^th^ percentile in a reference population of young female adults aged 20-29 years-old [34]. Open square bracket with an asterisk (*) represents significant difference between two groups at an alpha value of 0.05.

## Data Availability

The datasets generated during and/or analyzed during the current study are available from the corresponding author on reasonable request.

## Abbreviations

IBEM: Inborn error of metabolism
DXA: Dual-energy X-ray absorptiometry
HR-pQCT: High-resolution peripheral quantitative computed tomography
BMD: Bone mineral density
HPP: Hypophosphatasia
LSD: Lysosomal storage disorder
TtBMD: Total bone mineral density
CtBMD: Cortical bone mineral density
TbBMD: Trabecular bone mineral density
CtTh: Cortical thickness
CtPo: Cortical porosity
TbN: Trabecular number
TbTh: Trabecular thickness
TbSp: Trabecular separation
