# Supplementary figures and images for: Spectrum of microarchitectural bone disease in inborn errors of metabolism: a cross-sectional, observational study
Source: Orphanet J Rare Dis. 2020 Sep 16;15:251. doi: 10.1186/s13023-020-01521-6 (PMC7493311; doi:10.1186/s13023-020-01521-6)

## RADIUS

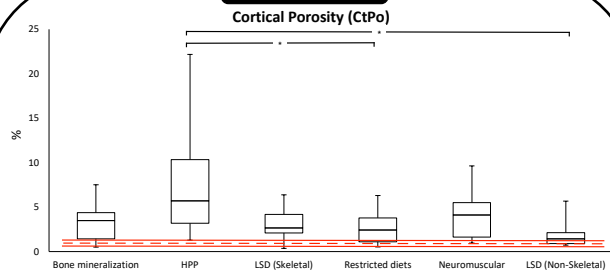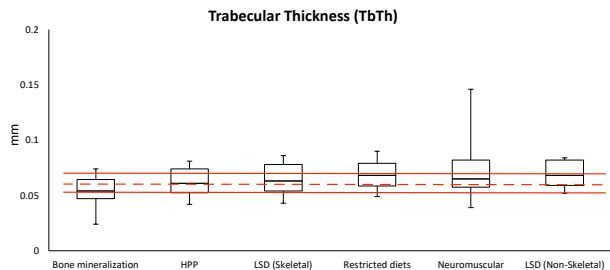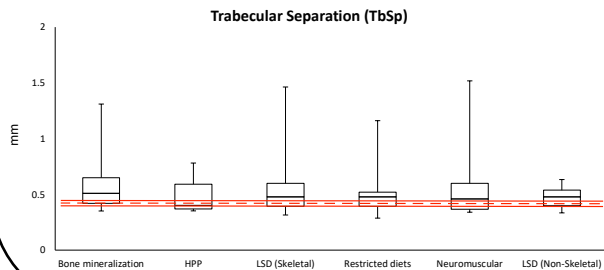

## TIBIA

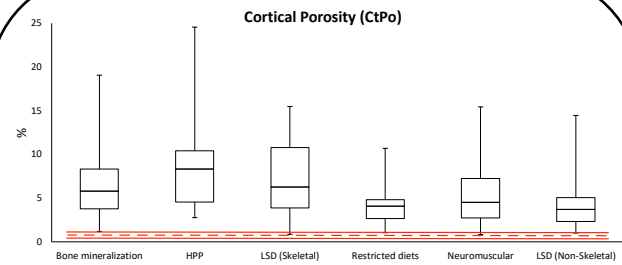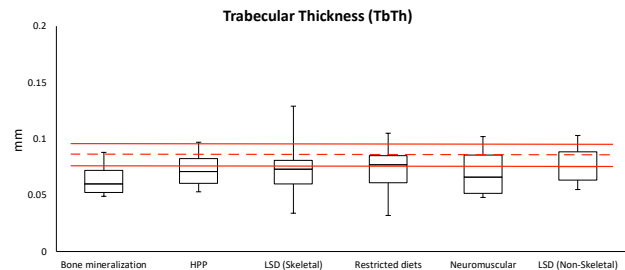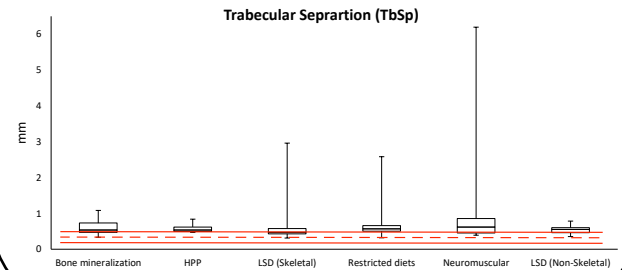

Supplement: Supplementary file 1 — Additional file 1: Supplemental Figure 1. Additional HR-pQCT measurements plotted. Radius and tibia analysis shown as box-and-whisker plot for HR-pQCT measured microarchitecture. Red lines, from top-to-bottom, represent 75th, 50th, and 25th percentile in a reference population of young female adults aged 20-29 years-old [34]. Open square bracket with an asterisk (*) represents significant difference between two groups at an alpha value of 0.05. [file 13023_2020_1521_MOESM1_ESM.pdf]
